# Evaluation of a Technology-Based Multifactorial Intervention for Improving Fall Risk: A Cluster-Randomized Controlled Trial

**DOI:** 10.21203/rs.3.rs-10412360/v1

**Published:** 2026-07-30

**Authors:** Chitra Banarjee, Kworweinski Lafontant, Jethro Raphael Suarez, Ania Lipat, Megha Parikh, Janet Lopez, Carla Leinbach, Rui Xie, Ladda Thiamwong

**Affiliations:** University of Central Florida College of Medicine; University of Central Florida College of Health Professions and Sciences; University of Central Florida Department of Mechanical and Aerospace Engineering; University of Central Florida College of Nursing; University of Central Florida College of Nursing; University of Central Florida College of Nursing; University of Central Florida College of Nursing; University of Central Florida College of Nursing; University of Central Florida College of Nursing

**Keywords:** older adults, fall prevention, dynamic balance, lower limb endurance, BTrackS, Timed Up and Go test, Chair Stand test

## Abstract

**Purpose:**

In the rapidly growing older adult demographic, falls are a critical issue as the leading cause of injury. Multifactorial interventions have been shown to improve fall risk, including physical function and fear of falling. This study evaluates the effect of the Physio-fEedback and Exercise pRogram (PEER), an 8-week multifactorial technology-based fall risk intervention.

**Methods:**

The longitudinal study included 387 community-dwelling, low-income older adults and was conducted at 4 timepoints at T1 (baseline), T2 (2 months), T3 (4 months), and T4 (6 months). The Timed Up and Go Test, 30-second chair stand test, and BTrackS Score were used as measures of physical function (dynamic balance, lower limb endurance, and static balance, respectively). The PEER intervention consisted of an 8-week technology-based physio-feedback, cognitive reframing, and group-based and at-home exercises (NCT05778604).

**Results:**

The PEER intervention mitigated time-related declines in dynamic balance post-intervention (*p*<0.001) and subsequent follow-up (*p*=0.005). Lower limb endurance and static balance did not show significant improvements.

**Conclusion:**

The intervention group showed sustained improvement of dynamic balance, indicating effectiveness of PEER after conclusion of the intervention. However, the lack of significant differences in the other fall risk metrics may be indicative of insufficient duration and/or intensity of the intervention. This study demonstrates both potential for the PEER technology-based mind-body fall prevention for improving dynamic balance and gaps in targeting measures of fall risk.

**Trial Registration:**

The trial was registered at ClinicalTrials.gov on March 21, 2023 (NCT05778604).

## Key summary points

**Aim:**

Evaluate the effect of the Physio-fEedback and Exercise pRogram (PEER), an 8-week multifactorial technology-based fall risk intervention, on improving fall risk measures. PEER integrates group-based and at-home exercises with physio-feedback and cognitive reframing to address physical and psychological risk factors.

**Findings:**

The PEER program decreased the time-dependent decline in dynamic balance immediately after the intervention and during follow-ups over 6 months. However, lower limb endurance and static balance did not improve after the intervention. The intervention group demonstrated high adherence to group and home exercises.

**Message:**

While PEER demonstrated a protective effect on dynamic balance, the intervention may need to increase exercise intensity and cognitive reframing implementation to elicit similar responses in other fall risk measures.

## Introduction

Falls are the leading cause of unintentional injury in older adults (aged 65 years and older) [1]. There are several types of fall prevention interventions that aim to reduce fall risk, including exercise, pharmaceutical, and environmental, as well as multifactorial interventions, that simultaneously focus on two or more fall risk factors, such as balance and fear of falling [2]. The United States Preventive Services Task Force (USPSTF) recommends multifactorial and exercise interventions, which have both been shown to result in improvements in fall-related outcomes, including number of falls and type of injury following a fall [2, 3].

Traditional fall risk interventions, however, are often conducted without providing participants with individualized feedback that promotes awareness and understanding of their own fall risk status. Feedback-based technology has been shown to improve physical and cognitive function in older adults [4, 5]. Cognitive behavioral therapy strategies, including cognitive reframing, goal setting, and motivational interviewing, have also proven useful in reducing fear of falling by disrupting a fear avoidance cycle [6–9]. Interventions that combine objective physiological assessments with personalized physio-feedback and cognitive strategies may help bridge the gap between fall risk identification and behavior change by increasing motivation, self-efficacy, and engagement in fall prevention activities [9, 10].

To address the limitations of current fall interventions, our team developed the PhysiofEedback Exercise Program (PEER) - an 8-week intervention consisting of technology-based physio-feedback, cognitive reframing, and a combined group- and home-based exercise program by a trained coach. While other multifactorial fall prevention interventions consider different aspects of fall risk, including balance performance and fear of falling (FoF), the physio-feedback provided in PEER encourages older adults’ involvement in the intervention. Previous pilot work by our group has demonstrated improvements in physical activity and handgrip strength after the PEER intervention [11–13]. While these measures play an important role in determining one’s fall risk, additional measures of physical function, such as dynamic balance, lower limb endurance, and postural sway are often considered stronger predictors of fall risk [14, 15].

Dynamic balance as measured by the Timed Up and Go (TUG) test is a standardized measure of mobility and gait that has shown specificity for identifying individuals at high fall risk [16]. Given the multifactorial nature of fall risk, interventions often utilize the TUG in conjunction with other measures of physical function, including lower limb endurance as measured by the 30-second chair stand test (30CST) and static balance as measured by the postural sway path length from the BTrackS balance plate [17–19]. Previous work examined the static balance performance of the participants included in this study following participation in PEER and reported no significant changes [13]. However, that investigation was exploratory in nature and focused on various spatial, temporal, and directional characteristics of postural sway rather than the BTrackS score. Although these metrics provide a comprehensive understanding of an individual’s static balance performance, the BTrackS score offers a more clinically relevant interpretation of intervention-related changes in fall risk because established normative ranges are directly associated with fall risk (as seen from the output of the Balance and Fall Risk protocol of the BTrackS). The BTrackS score was included in analysis for the current study to provide a more clinically interpretable assessment of balance-related changes.

Here, we present findings on the effect of PEER on fall risk factors in a large cohort of older adults at four timepoints over six months. We hypothesized that the PEER intervention would lead to improvement in static and dynamic balance and lower limb endurance.

## Methods

### Clinical Trial Design

The effects of the PEER intervention were evaluated through an intention-to-treat, single-blinded, parallel, two-arm cluster-randomized controlled trial (RCT) funded by the National Institute on Minority Health and Health Disparities (R01MD018025) and pre-registered on ClinicalTrials.gov (NCT05778604) and posted on March 21, 2023. Data was collected at baseline (T1) with outcomes measured immediately after program completion (T2) and follow-ups at 3-months (T3) and 6-months (T4) to evaluate post-intervention and follow-up changes in physical function and fall risk. The trial has been reported in accordance with the CONSORT 2025 guidelines.

#### Ethics.

All study protocols were approved by the University of Central Florida Institutional Review Board (STUDY00003206), carried out in accordance with the Declaration of Helsinki, and have been previously published elsewhere [20]. All participants provided written informed consent prior to participation.

#### Trial Setting and Recruitment.

The trial was conducted in low-income independent living communities/units in Orlando, FL between April 2023 to August 2025. Participants were recruited from 18 independent living communities and senior centers in Orlando, FL through flyer distribution, word-of-mouth, and community partners facilitating our introduction.

#### Eligibility.

The inclusion criteria comprised of (1) age of 60 years or older, (2) being able to walk with or without an assistive device (but without the assistance of another person), (3) fluency in English or Spanish, and (4) living in their own homes or apartments. The exclusion criteria were as follows: (1) a medical condition precluding exercise such as uncontrolled cardiac disease (shortness of breath or feeling pressure, squeezing, burning, or tightness when exercising), or (2) currently receiving treatment from a rehabilitation facility. Eligible participants were assigned to either the intervention or control group through cluster randomization design, with senior living communities/sites serving as clusters.

#### Intervention.

**Figure 1:**
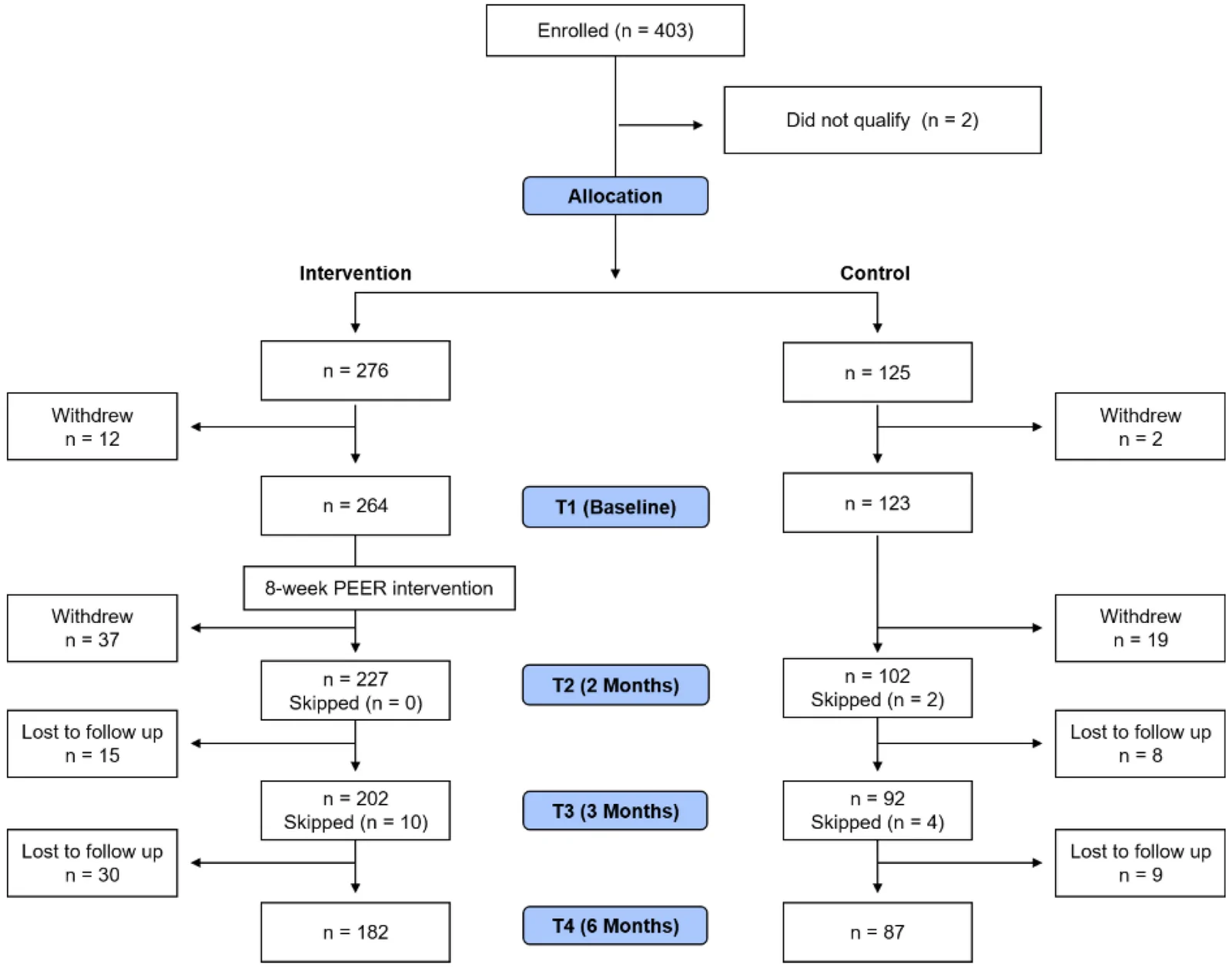
Flow of participants over the four timepoints.

The intervention group received the 8-week PEER, which included (1) technology-based physio-feedback (Fig. 1) via portable BTrackS balance assessments; (2) cognitive reframing; and (3) peer-led exercises focusing on balance, strength training, and incorporating exercises into daily activities, as described previously [20]. Cognitive reframing was conducted as a discussion with a researcher regarding participants’ static balance performance and fall risk pre-intervention and post-intervention. Participants engaged in weekly 1-hour group exercise sessions for 8 weeks. Sessions were delivered by trained instructors with characteristics similar to those of participants (e.g., age or cultural background) and were supervised by a certified exercise physiologist. Static stretching, low-load resistance exercises, and seated/standing balance exercises were performed during each group exercise session. Home-based exercises were self-completed each week and guided by a research team–developed exercise booklet. The booklet included exercises similar to those performed during the group sessions, focused on upper and lower body strengthening as well as static and dynamic balance. Participants were instructed to complete a minimum of 60 minutes of exercises from the booklet per week.

The control group received U.S. Centers for Disease Control and Prevention informational pamphlets about ways to prevent falls, how to check for fall safety at home, and postural hypotension. The control group were encouraged to discuss fall prevention with their primary care provider and continue their regular activities for 6 months. At the conclusion of the study, each control site was offered the PEER intervention.

### Outcomes

The primary outcomes for the randomized controlled trial were dynamic balance, FoF, and physical activity (PA). However, this study presents the results regarding dynamic balance, lower limb endurance, and static balance. Exercise adherence and occurrence of falls during the study period were additionally assessed as secondary outcomes. Other trial outcomes will be reported separately. All physical function assessments were preceded by instruction and demonstration of the assessment by trained research team members.

#### Dynamic Balance.

Dynamic balance was assessed using the TUG test [21]. Participants were instructed to stand up from a chair, walk 3 meters, turn around a taped line, walk back to the chair, and sit down again. The measure of dynamic balance was recorded as the time in seconds that the participants took to complete the assessment.

#### Lower Limb Endurance.

Lower limb endurance was assessed using the 30CST [18]. Participants were instructed to stand up from a chair without using their arms as many times as possible within 30 seconds. The measure of lower limb endurance was recorded as the number of chair stands the participant completed.

#### Static Balance.

Static balance (center-of-pressure postural sway path length; i.e., BTrackS Score) quantified using the previously validated BTrackS (Balance Tracking Systems, Inc. San Diego, CA), consisting of a balance plate and BTrackS software (Version 7.5.5). Participants completed one 20-second trial for familiarization, in which they were instructed to stand on the balance plate with their feet 30cm apart, hands on hips, and eyes closed. After this practice trial, three additional 20-second trials were conducted and averaged to obtain the final static balance score in centimeters [22]. The BTrackS has been validated for reliability and validity using intraclass correlation coefficients and Pearson correlations [23].

#### Exercise Adherence.

Exercise adherence was evaluated separately for in-class and at-home components of the PEER intervention. Attendance was taken at each exercise class and participants self-reported at-home adherence via weekly paper exercise logs, which they turned in at the start of each group exercise class. In-class adherence was calculated as the percentage of the 8 group exercise classes attended by each participant over the intervention period. At-home adherence was calculated as the percentage of the 8 weeks that the participant completed at least 60 minutes of specified exercises at home.

#### Occurrence of Falls.

Occurrence of falls was assessed by phone call using a monthly fall incidence log for each participant. A fall was defined as any event that resulted in coming to rest inadvertently on the ground or floor or other lower level, as defined by the World Health Organization [24].

### Statistical Methods

Sample size. The sample size estimate for the RCT was based on the primary outcomes of TUG time, 30CST repetitions, and BTrackS score, as described previously [20]. Based on previous studies, medium effect size Cohen’s f of 0.25, intraclass correlation (ICC) of 0.8, and a significance level of 0.05 were used for sample size calculation. A sample size of 120 participants per arm was needed to reach 80% statistical power to detect the difference in repeated measures between PEER and control groups.

#### Randomization.

Participants were grouped by site, and each site was randomized as either an intervention site (site n = 11) or a control site (site n = 6) as described previously [25].

#### Statistical Analysis.

All data were stored in a Research Electronic Data Capture (REDCap) database managed by the University of Central Florida [26, 27]. Mixed effects (multilevel) modeling was conducted in R statistical software (version 4.1.2, R Core Team, Vienna, Austria) with statistical significance level set at 0.05. The Shapiro–Wilk test was utilized to determine if continuous variables followed normal distributions, and log-transformations were applied to continuous outcomes with non-normal distributions and indicated in tables. Linear mixed effects regression models were fitted using the lmer function in the “lme4” package. Linear mixed-effects models used all available observations and accommodated missed follow-up visits; therefore, participants with skipped timepoints remained in the analyses without imputation of missing outcome data. The models were optimized using the “bobyqa” optimizer. Models employed restricted maximum likelihood (REML) estimation method for parameterization [28]. Intra-class coefficients (ICC) were computed using the “sjstats,” which utilizes the unconditional ICC for a random intercept model based on Wu et al 2012 for mixed effects models and the Wald method for calculating degrees of freedom [29, 30]. Observed power was estimated using simulation-based methods (package “simr”) for key predictors in each fitted model. Power estimates reflect the sensitivity of the model to detect effects of the magnitude observed in the data [31]. Risk ratios were calculated by unconditional maximum likelihood estimation using the riskratio function in the “epitools” package [32]. The final models included fixed effects of group (intervention compared to control as the reference group), timepoint (baseline, with follow-up at 2, 3, and 6 months), age, and sex, an interaction between group and timepoint, and random effects of participant and site.

## Results

### Participants

Of the 403 participants recruited, 387 were included in the current analyses. Participants were excluded if they did not meet inclusion criteria. The participants were 74.1 ± 6.93 years old, 87.8% female, and of diverse race and ethnicity (Non-Hispanic White 20.2%, Hispanic 24.8%, African American 47.3%, Asian 5.68%, and Other 2.07%). Table 1 describes baseline characteristics for both groups, and Fig. 1 describes the participant flow over the four timepoints.

### Primary Outcomes

#### Dynamic Balance.

There were significant main effects of group, time, age, and race, and a significant interaction of group × time post-intervention and over the 6-month follow-up (*p* < 0.05) (Table 2). In Model 1A, the intervention group had worse TUG scores overall (β = 0.35, *p* = 0.008). Dynamic balance worsened with time (β = 0.20, *p* < 0.001), age (β = 0.02, *p* < 0.001), and “Other” race/ethnicity (β = 0.28, *p* = 0.016), with increased TUG score reflecting worse dynamic balance. The intervention mitigated the time-dependent decline of dynamic balance after 8 weeks over time, as shown by the significant interaction (β = −0.21, *p* < 0.001). These post-intervention effects (Model 1A) held steady across the 6-month period (Model 1B), with worse performance as time (β = 0.06, *p* < 0.001) and age progress (β = 0.02, *p* < 0.001) and less worsening over time for the intervention group (β = −0.04, *p* = 0.005). Power analysis using likelihood ratio test revealed high power for the post-intervention interaction effect (99.67% with 95% confidence interval of 98.80%, 99.96%) and when including follow-ups (78.00% with 95% confidence interval of 68.61%, 85.67%).

#### Lower Limb Endurance.

Lower limb endurance, as measured by the 30CST did not significantly change post-intervention, but improved over the four timepoints (β = 0.42, *p* < 0.001). Performance at both timepoints was not affected by intervention or sex (*p* > 0.05) (Table 3). At both timepoints, age (Model 2A: β = −0.15, *p* < 0.001, Model 2B: β = −0.16, *p* < 0.001) and race (Model 2A: β = −3.19, *p* = 0.043, Model 2B: β = −3.35, *p* = 0.042) were significant main effects, such that endurance decreased with increased age and “Other” race (Table 3).

#### Static Balance.

Static balance, as measured by BTrackS score, did not significantly change over the four timepoints (p > 0.05) (Table 4). An increased BTrackS score was significantly associated with increased age (Model 3A: β = 0.02, *p* < 0.001, Model 3B: β = 0.02, *p* < 0.001) and the male sex (Model 3A: β = 0.16, *p* = 0.016, Model 3B: β = 0.17, *p* = 0.009), while a decreased BTrackS score immediately after the intervention and at follow-ups was significantly associated with the African American race (Model 3A: β = −0.19, *p* = 0.005, Model 3B: β = −0.17, *p* = 0.012) and Hispanic ethnicity (Model 3A: β = −0.23, *p* = 0.001, Model 3B: β = −0.21, *p* = 0.001) (Table 4).

### Secondary Outcomes

#### Exercise Adherence.

Adherence to the group exercise classes was 83.8 ± 18.6%. Adherence to the at-home exercises was 75.5 ± 25.3%.

#### Occurrence of Falls.

Over the course of the study, of the 387 participants, 56 (14.5%) older adults experienced falls between both the control (n = 22, 17.9%) and intervention (n = 34, 12.9%) groups. Chisquare test of independence did not reveal a statistically significant difference by group (*p* = 0.251). The risk of falls did not differ significantly between the control and intervention groups (risk ratio = 0.72, 95% CI [0.44, 1.18], *p* = 0.192).

## Discussion

In this study, we evaluated a novel mind-body intervention to improve fall risk in 387 older adults. The primary outcomes included dynamic balance, lower limb endurance and static balance. Our results suggest that the intervention resulted in short- and long-term improvements in dynamic balance, but not lower limb endurance or static balance.

Dynamic balance performance in the current study was defined as the time to complete the TUG test in seconds. While literature shows limitations in the predictive ability of the TUG for future falls, it is well established as an assessment for quantifying balance deficits [33–37]. Using this measure, we observed significant main effects of group and time, indicating that the intervention group had worse overall TUG scores, and both groups worsened over time. However, the intervention ameliorated this time-related worsening of dynamic balance. This protective effect was held both directly after the intervention (Table 2A) and four months after the intervention (Table 2B), as indicated by the high-powered effect post-intervention and low-moderate effect in subsequent follow-ups we observed. As increased age was also associated with worse TUG performance, the ability of PEER to slow the rate of decline in dynamic balance function holds clinical relevance and suggests that providing physio-feedback in conjunction with group exercise and cognitive reframing is a promising intervention for improving fall risk in older adults

The PEER intervention did not, however, result in a significant improvement for the other physical function measures of fall risk. Other studies have shown that both TUG and the 30CST were responsive to 8-week group-based and feedback-based interventions [38–40]. Additionally, both functional tests have similar discriminative ability for physical function over time [17]. Although 30CST performance improved significantly over time, no intervention-related effect was observed (Table 3). The null effect of the intervention on lower limb endurance may result from decreased measurement sensitivity (number of repetitions compared to time to complete) compared to more robust measures such as sit-to-stand muscle power [41]. Similarly, the BTrackS score, which is a measure of center-of-pressure path length, did not improve at either timepoint comparison (Table 4). However, this measure has shown improvements in other fall prevention interventions, but these typically have longer durations of at least 3 months or smaller sample sizes [42–46]. Taken together, the results in this large sample suggest that the intensity and duration of the intervention were not effective for improving the other physical function measures.

This study presented some limitations in the design, implementation, and evaluation of the intervention. The intervention consisted of group- and home-based exercises for 8 weeks and two cognitive reframing sessions, resulting in low intensity and limited duration that may have impacted the non-significant improvements in the 30CST and BTrackS. However, older adults had above average adherence to the intervention in-class and at-home, which may have contributed to improvements in dynamic balance [47]. Additionally, older adults were evaluated at baseline, after the intervention, and two additional time points at three and six months, for revealing post-intervention and follow-up effects. The duration of the study presents a limitation in evaluating long-term effects beyond six months. Future work should investigate increasing the intensity and duration of the intervention and expanding the follow-up timepoints. Additionally, evaluation of dynamic balance as time to complete TUG subtasks, such as turning, rather than total time to complete the TUG, may provide insight as to the mechanism of PEER in improving this outcome [48].

## Conclusion

The PEER intervention provides an innovative technology-based fall prevention program for community-dwelling older adults that targets multifactorial fall risk. This study evaluated the effects of PEER on important fall risk factors in community settings. The results demonstrate that this intervention attenuated declines in dynamic balance, but they also show gaps that may improve PEER in future work, including increasing the duration and intensity of the intervention as well as improving the implementation of cognitive reframing.

## Supplementary Material

This is a list of supplementary files associated with this preprint. Click to download.

- floatimage1.png

## Figures and Tables

**Table 1:** Baseline characteristics of included participants. TUG = Timed Up and Go, 30CST = 30 second chair stand test, FoF = fear of falling

Variable	Control	Intervention
Age (years)	74.4 ± 7.54	74.35 ± 6.88
Female n (%)	106 (86.18%)	233 (88.59%)
TUG score (seconds)	10.9 ± 7.76	11.29 ± 4.79
30CST score (repetitions)	11.55 ± 4.32	11.97 ± 4.06
BTrackS score (cm)	35.68 ± 22.14	31.77 ± 17.86

**Table 2:** Effects of Group and Time on dynamic balance. In this model, control is the reference group, and female is the reference group. Bolded *p*-values are significant. TUG refers to the Timed Up and Go test, measured in seconds; ICC refers to Intraclass Correlation Coefficient.

	Log (TUG Score)					
	Model 1A (T1 vs T2)			Model 1B (T1-T4)		
*Predictors*	*Estimate*	*CI*	*p*	*Estimate*	*CI*	*p*
(Intercept)	0.48	0.06–0.89	**0.024**	0.63	0.28–0.98	**< 0.001**
Group [Intervention vs Control]	0.35	0.09–0.61	**0.008**	0.11	−0.10–0.31	0.312
Time	0.20	0.13–0.27	**< 0.001**	0.06	0.04–0.09	**< 0.001**
Age	0.02	0.02–0.02	**< 0.001**	0.02	0.02–0.02	**< 0.001**
Sex [Male vs Female]	−0.04	−0.13–0.06	0.455	−0.03	−0.11–0.06	0.511
Race/Ethnicity [Asian]	0.01	−0.17–0.18	0.947	0.03	−0.12–0.18	0.725
Race/Ethnicity [African American]	0.11	−0.01–0.24	0.068	0.13	0.02–0.23	**0.016**
Race/Ethnicity [Hispanic]	−0.05	−0.16–0.06	0.350	−0.02	−0.11–0.08	0.716
Race/Ethnicity [Other]	0.28	0.05–0.51	**0.016**	0.32	0.12–0.52	**0.001**
Group × Time	−0.21	−0.30 – −0.13	**< 0.001**	−0.04	−0.06 – −0.01	**0.005**
**Random Effects**						
σ^2^	0.07			0.06		
τ_00 Participant_	0.05			0.05		
τ_00 Site_	0.04			0.03		
ICC	0.57			0.58		
N _Participant_	380			380		
N _Site_	17			17		
Observations	704			1281		
Marginal R^2^ / Conditional R^2^	0.128 / 0.624			0.144 / 0.643		

**Table 3:** Effects of Group and Time on lower limb endurance. In this model, control is the reference group, and female is the reference group. Bolded *p*-values are significant. 30CST refers to the 30-second chair stand test, measured in completed repetitions; ICC refers to Intraclass Correlation Coefficient

	30CST Score					
	Model 2A (T1 vs T2)			Model 2B (T1-T4)		
*Predictors*	*Estimate*	*CI*	*p*	*Estimate*	*CI*	*p*
(Intercept)	21.70	16.76–26.64	**< 0.001**	22.40	17.36–27.44	**< 0.001**
Group [Intervention vs Control]	−0.09	−2.00–1.83	0.928	0.38	−1.25–2.02	0.645
Time	0.44	−0.19–1.07	0.173	0.42	0.21–0.64	**< 0.001**
Age	−0.15	−0.21 – −0.09	**<0.001**	−0.16	−0.22 – −0.10	**<0.001**
Sex [Male vs Female]	0.66	−0.57–1.89	0.294	0.98	−0.32–2.28	0.138
Race/Ethnicity [Asian]	1.18	−0.86–3.23	0.257	1.09	−1.03–3.21	0.314
Race/Ethnicity [African American]	0.08	−1.31–1.47	0.908	−0.43	−1.84–0.98	0.552
Race/Ethnicity [Hispanic]	0.76	−0.58–2.10	0.267	0.78	−0.60–2.16	0.267
Race/Ethnicity [Other]	−3.19	−6.27 – −0.10	**0.043**	−3.35	−6.58 – −0.11	**0.042**
Group × Time	0.47	−0.29–1.22	0.224	0.12	−0.14–0.37	0.373
**Random Effects**						
σ^2^	4.65			4.89		
τ_00 Participant_	11.82			14.24		
τ_00 Site_	1.35			1.15		
ICC	0.74			0.76		
N _Participant_	362			367		
N _Site_	17			17		
Observations	656			1197		
Marginal R^2^ / Conditional R^2^	0.080 / 0.760			0.087 / 0.780		

**Table 4:** Effects of Group and Time on static balance. In this model, control is the reference group, and female is the reference. Bolded *p*-values are. ICC refers to the intra-class correlation coefficient.

	Log (BTrackS Score)					
	Model 3A (T1 vs T2)			Model 3B (T1-T4)		
*Predictors*	*Estimate*	*CI*	*p*	*Estimate*	*CI*	*p*
(Intercept)	2.47	1.97–2.97	**< 0.001**	2.43	1.95–2.91	**< 0.001**
Group [Intervention vs Control]	−0.07	−0.22–0.08	0.377	−0.10	−0.23–0.03	0.144
Time	−0.02	−0.07–0.04	0.559	−0.01	−0.03–0.00	0.129
Age	0.02	0.01–0.02	**< 0.001**	0.02	0.01–0.02	**< 0.001**
Sex [Male vs Female]	0.16	0.03–0.29	**0.016**	0.17	0.04–0.30	**0.009**
Race/Ethnicity [Asian]	−0.09	−0.30–0.12	0.397	−0.05	−0.25–0.15	0.637
Race/Ethnicity [African American]	−0.19	−0.32 – −0.06	**0.005**	−0.17	−0.30 – −0.04	**0.012**
Race/Ethnicity [Hispanic]	−0.23	−0.37 – −0.10	**0.001**	−0.21	−0.34 – −0.08	**0.001**
Race/Ethnicity [Other]	−0.21	−0.52–0.10	0.188	−0.20	−0.50–0.10	0.181
Group × Time	−0.01	−0.08–0.05	0.642	0.01	−0.01–0.03	0.518
**Random Effects**						
σ^2^	0.04			0.03		
τ_00 Participant_	0.15			0.15		
τ_00 Site_	0.00			0.01		
ICC	0.81			0.83		
N _Participant_	383			383		
N _Site_	17			17		
Observations	711			1293		
Marginal R^2^ / Conditional R^2^	0.116 / 0.836			0.116 / 0.846		

## Data Availability

The deidentified data for this study, as well as the statistical processing code, is publicly available at https://osf.io/5eq4y/overview.

## Abbreviations

30CST: 30-Second Chair Stand Test
BTrackS: Balance Tracking System
CI: Confidence Interval
FoF: Fear of Falling
ICC: Intraclass Correlation Coefficient
PA: Physical Activity
PEER: Physio-Feedback Exercise Program
RCT: Randomized Controlled Trial
REDCap: Research Electronic Data Capture
REML: Restricted Maximum Likelihood
T1: Baseline Assessment Timepoint
T2: Post-Intervention Assessment Timepoint
T3: 3-Month Follow-Up Assessment Timepoint
T4: 6-Month Follow-Up Assessment Timepoint
TUG: Timed Up and Go Test
USPSTF: United States Preventive Services Task Force
